# (De)glutamylation and cell death in *Leishmania* parasites

**DOI:** 10.1371/journal.pntd.0007264

**Published:** 2019-04-24

**Authors:** Louise Basmaciyan, Derrick R. Robinson, Nadine Azas, Magali Casanova

**Affiliations:** 1 Aix Marseille Univ, IRD, AP-HM, SSA, VITROME, Marseille, France; 2 IHU-Méditerranée Infection, Marseille, France; 3 Laboratoire MFP CNRS UMR-5234, France; McGill university, CANADA

## Abstract

Trypanosomatids are flagellated protozoan parasites that are very unusual in terms of cytoskeleton organization but also in terms of cell death. Most of the Trypanosomatid cytoskeleton consists of microtubules, forming different substructures including a subpellicular corset. Oddly, the actin network appears structurally and functionally different from other eukaryotic actins. And Trypanosomatids have an apoptotic phenotype under cell death conditions, but the pathways involved are devoid of key mammal proteins such as caspases or death receptors, and the triggers involved in apoptotic induction remain unknown. In this article, we have studied the role of the post-translational modifications, deglutamylation and polyglutamylation, in *Leishmania*. We have shown that *Leishmania* apoptosis was linked to polyglutamylation and hypothesized that the cell survival process autophagy was linked to deglutamylation. A balance seems to be established between polyglutamylation and deglutamylation, with imbalance inducing microtubule or other protein modifications characterizing either cell death if polyglutamylation was prioritized, or the cell survival process of autophagy if deglutamylation was prioritized. This emphasizes the role of post-translational modifications in cell biology, inducing cell death or cell survival of infectious agents.

## Author summary

*Leishmania* are unique unicellular organisms in terms of cytoskeleton organization and mechanisms of cell death. For example, the major cytoskeletal components of these parasites are microtubules, which form a subpellicular corset. In terms of cell death, an apoptotic phenotype has been characterized in *Leishmania* but the pathways remain unknown, being devoid of key mammal cell death proteins. In a previous article, we demonstrated that the cytoskeleton of this parasite is extensively glutamylated but, paradoxically, overexpression or inhibition of polyglutamylase expression have limited visible cellular consequences. In this manuscript, we have highlighted the link between polyglutamylation and *Leishmania* cell death, suggesting the importance of the polyglutamylation/deglutamylation balance in this parasite. Further, we have identified, for the first time in *Leishmania*, deglutamylases, among which one that, in an original manner, deglutamylates glutamates at branching points but also long glutamate side chains. This work emphasizes the role of post-translational modifications as essential regulators of protein function, not only of mammal cells such as neurons or ciliated/flagellated cells, but also of infectious agents. This work suggests an important and discernible “live or die”—“cell death or autophagy” balance pathway and the conceptual mechanism that is involved in cellular decision making.

## Introduction

Microtubules are key components of the eukaryotic cytoskeleton that dynamically assemble from heterodimers of α- and β-tubulin, and whose structure and protein sequence are highly conserved in evolution. Microtubules are involved in intracellular transport, organelle positioning, cell shape, mitosis or cell mobility. Two different mechanisms can generate microtubule diversity, explaining their large variety of cellular functions: the expression of different α- and β-tubulin genes, referred to as tubulin isotypes, and the generation of post-translational modifications (PTM) on their C-termini (acetylation, phosphorylation, polyglutamylation, polyglycylation, palmitoylation, polyamination and detyrosination) [2,3]. PTMs mark subpopulations of microtubules and selectively affect downstream microtubule-based functions [4]. In this way, the tubulin modifications generate a “code” called the “tubulin code”, linked to the nature, length and spacing patterns of these modifications, that can be read by microtubule-associated proteins in a manner analogous to how the histone code directs diverse chromatin functions [4]. Among microtubule modifications, polyglutamylation has recently been documented. It generates glutamate side chains of variable length on the gamma-carboxyl group of glutamate residues within the primary sequence of the target protein, essentially α- and β-tubulins [5]. Polyglutamylation may help stabilise or conversely destabilise microtubules; it may also affect processes such as the interaction of microtubules with kinesins, microtubule-associated proteins or microtubule-severing factors through a modulation of affinity depending on the polyglutamate chain length and positioning [2,6–9]. Polyglutamylation is generated by members of the Tubulin Tyrosine Ligase-Like (TTLL) family [10], while deglutamylation is mediated by members of the cytosolic carboxypeptidase (CCP) family [11,12]. Each polyglutamylase displays defined reaction preferences, for modifying the α- or β-tubulin, for generating short or long side chains and for initiating or elongating the chain [12,13]. Polyglutamylases can also modify many other substrates than tubulins, such as nucleocytoplasmic shuttling proteins [14].

*Leishmania* are kinetoplastids and are flagellated parasitic protozoa of the Trypanosomatid family. Microtubules are highly abundant constituents of the Trypanosomatid cytoskeleton [15]. They are present in four sub-structures: the mitotic spindle, the flagellar axoneme, the basal body of the flagellum and the sub-pellicular “corset”. This corset is exclusively made of a dense network of microtubules that are cross-linked to each other and to the plasma membrane, forming a helical pattern along the long axis of the cell [16]. The cytoskeleton is responsible for cell shape and plays a major role in events such as positioning of organelles, mitosis and cytokinesis [17]. Our published data demonstrated that *Leishmania* microtubules are intensely glutamylated at all stages of the cell cycle and identified four proteins which appeared to be involved in microtubule polyglutamylation, using *in vitro* activity assays: LmTTLL4A and LmTTLL6B that proved clearly to be active enzymes, whereas LmTTLL4C and LmTTLL6A had only slight activity on the substrates tested [18]. The results from that work underline that, paradoxically, in view of the importance of tubulins in these organisms, and of their extensive glutamylation, the inhibition of most TTLL has no effect on cell growth or cell cycle of *Trypanosoma brucei* procyclic forms, a parasite from the same Trypanosomatid family. Furthermore, for the moment, no deglutamylase has been identified in Trypanosomatids.

Under a variety of stress stimuli including nitric oxide or reactive oxygen species produced by the host, hydrogen peroxide or leishmanicidal drugs such as amphotericin B, curcumin, miltefosine or pentamidine, apoptosis-like morphological and biochemical features have been described in *Leishmania*, among which growth inhibition, cell rounding up, cell shrinkage, mitochondrial depolarization or TUNEL-positivity [19–25]. Since apoptosis is defined by its morphology [26], we can talk about apoptosis in this parasite. In *Leishmania*, it has been demonstrated that cell death is paradoxically essential for successful survival of the population and for parasite infectivity [27]. Indeed, apoptosis allows regulating the parasite cell density in the host to avoid hyperparasitism [27]. It allows the fittest cells to survive and to be selected, unfit cells being eliminated [28]. It also modulates host immunity [27]. Despite the evidence for apoptosis in *Leishmania*, very little is known about the cell death pathways and the implicated executioner proteins. Indeed, essential proteins involved in mammalian apoptosis, such as death receptors and caspases, are apparently not encoded in the genome of *Leishmania* [29] and the existence of pro-apoptotic molecules is still controversial [30].

The work presented in this article aims at defining the link betweeen PTMs, deglutamylation and polyglutamylation, and cell death in *Leishmania*. We demonstrated that polyglutamylases were overexpressed during cell death and that overexpression of some polyglutamylases induced *Leishmania* apoptosis. Conversely, overexpression of deglutamylases inhibited *Leishmania* regulated cell death (RCD). We hypothesized that autophagic stimuli such as serum deprivation induce deglutamylases overexpression and so *Leishmania* survival through autophagy, rendering the balance between polyglutamylation/deglutamylation essential for *Leishmania* homeostasis: imbalance induces either cell death or cell survival. This work corroborates the importance of PTM as cytoskeleton regulators, already identified in several pathologies, but here emphasized in an infectious disease.

## Methods

### Parasites

*L*. *major* ‘Friedlin’ promastigotes (MHOM/IL/81/Friedlin) were grown in Schneider’s *Drosophila* medium (Life Technologies, Saint-Aubin, France) supplemented with 100U/mL penicillin, 100μg/mL streptomycin, 2mM glutamin and 20% heat inactivated fetal calf serum (FCS) (Life Technologies) at 26°C.

### Molecular constructs

The gene encoding the deglutamylases CCP5A (*LmjF*.*34*.*2810*) and CCP5B (*LmjF*.*36*.*4030*) were PCR-amplified from *L*. *major* genomic DNA. The PCR products were cloned into pGEM-T-Easy (Promega, Madison, WI, USA) before digestion by MfeI and HpaI restriction enzymes and insertion into the expression vectors pTH6cGFPn and pTH6nGFPc previously digested by the same enzymes (kind gift from Patrick Bastien, Montpellier University)[31]. These constructions allowed, after *Leishmania* transfection, the episomal expression of CCP5A or CCP5B fused to the Green Fluorescent Protein (GFP) in N-terminal (pTH6cGFPn vector) or C-terminal (pTH6nGFPc vector). The reading frame of the recombinant protein was checked by sequencing.

### Transfection procedure

Logarithmic *L*. *major* promastigotes were harvested by centrifugation at 600xg for 10min, washed once in sterile PBS and resuspended at 3x10^7^cells/mL in 100μL of Human T Cell Nucleofector solution (Lonza, Basel, Switzerland). Cells were transferred to Amaxa electroporation cuvettes maintained at 4°C containing 10μg of DNA. Cells were then electroporated with the program U-033 on the Nucleofector machine (Amaxa GmbH, Cologne, Germany). Following electroporation, cells were incubated overnight in their culture medium and transfectants were selected with 30μg/mL hygromycin B (Life Technologies).

### Induction of cell death and autophagy

Cell death was induced by harvesting logarithmic *L*. *major* cells by centrifugation at 600xg for 10min and incubating cells at 10^7^cells/mL in culture medium with 40μM miltefosine (Santa Cruz Biotechnology, Dallas, TX, USA) or 50μM curcumin (Sigma-Aldrich, Saint-Louis, MO, USA) for 24h.

For nutrient deprivation, logarithmic *L*. *major* cells, after harvesting, were washed once with sterile PBS and incubated at 10^7^cells/mL in a serum-deprived medium. Cell concentration was evaluated using a Thoma counting chamber.

### Determination of miltefosine and curcumin IC50

In order to determine the miltefosine and curcumin IC50, a MTT assay was carried out. Briefly, promastigotes in log-phase were incubated at an average density of 10^6^ parasites/mL in sterile 96-well plates with various concentrations of miltefosine dissolved in water or curcumin dissolved in ethanol (final concentration less than 0.5% v/v) incorporated in triplicate. Appropriate controls without any drug and with ethanol were added to each set of experiments. After a 72h incubation period at 26°C, parasite metabolic activity was determined. After the addition of MTT (0.5mg/ml in PBS, 20μl/well), plates were incubated for 4 h at 26°C. The reaction was stopped and the pellet dissolved by addition of 100μL of 10% SDS + 50% isopropanol. The absorbance was measured in a plate reader at 570nm. Inhibitory concentration 50% (IC50) was defined as the concentration of drug required to inhibit by 50% the metabolic activity of *Leishmania* compared to the control.

For determination of the optical density, the same protocol has been used. Indeed, 20μL of MTT 0.5mg/mL was added to 100μL of each sample in triplicate. It was incubated for 4h at 26°C before addition of 100μL of SDS/isopropanol and absorbance measure at 570nm in a plate reader.

### Immunofluorescence imaging

For cytoskeleton preparation, cells were washed in PBS, gently resuspended in PIPES 100mM pH 6.9, MgCl_2_ 1mM, Nonidet P-40 0.25%, washed in PBS and fixed in 4% paraformaldehyde (PFA) (4°C, 30 min). In the other cases, cells were directly fixed in PFA. Cells were then air-dried on microscope fluorescence slides after a PBS wash and the slides were mounted with SlowFade Gold antifade mountant with DAPI (Life Technologies). For immunofluorescence, cells were permeabilized 10min using 0.2% Triton X-100 in PBS after fixation, washed in PBS and incubated with the GT335 (1:10,000, Adipogen, San Diego, CA, USA), the PolyE (1:10000, kind gift from Carsten Janke, Curie Institure, Paris-Sud 11 University) or anti-α-tubulin (12G10, 1:500, kind gift from Carsten Janke) antibodies for 1h, followed by 45min with a goat anti-mouse Texas Red antibody (1:500, Life Technologies). After PBS wash, slides were mounted. Observations were done using a BX51 fluorescence microscope (Olympus, Rungis, France) and images acquired using the fluorescence imaging system Cell^A^ (Olympus). The maximum of GT335 and PolyE fluorescence was quantified using the Image J software.

### Immuno-electron microscopy

A mid-log phase *L*. *major* GFP-tagged CCP5A cell culture (5mL) was harvested, 1,000xg for 10min, washed in PBS (1,000xg) and resuspended in 500μL PBS. The cell suspension was placed on parafilm strips on a flat surface and glow-discharged, carbon and formvar coated, G200 nickel EM grids were floated onto the droplets for 5 min RT to adhere the cells to the grids. The droplets were then transferred onto 1% IGEPAL CA-630 (Sigma-13021) in PEME buffer (10min, RT)(2 mM EGTA, 1 mM MgSO4, 0.1 mM EDTA, 0.1 M piper-azine-N,N = -bis(2-ethanesulfonic acid)–NaOH (PIPES-NaOH), protease inhibitor cocktail, pH 6.9) and washed four times in PEME buffer. Grids were transferred to droplet containing 4% PFA in PBS for 10min. Fixed cytoskeletons were then neutralised 2 x 10min in 100mM glycine in PBS. Cytoskeletons were incubated with rabbit anti-GFP (Clontech, Saint-Germain-en-Laye, France), 1:100 in PBS+0.1% Tween 2h at RT. Grids were washed 3 x 10min in PBS and then incubated in a 50:50 mixture A and G 10nm gold particles (Electron Microscopy Sciences, Hatfield, PA, USA) diluted 1:20 in PBS. Grids were washed 3 x 10min in PBS, then fixed in 2.5% glutaraldehyde in PBS for 5min, washed in PBS 2 x 5min, air dried and negatively stained in 5μL Nanovan. Images were viewed and recorded on a Philips Technai 12 TEM.

### TUNEL

To detect DNA double-strand breaks, we applied the TUNEL test using the *in situ* cell death detection kit, fluorescein (Roche, Meyla, France). Cells were fixed with PFA 4%, adhered onto an immuno-slide and permeabilized with a 0.1% triton X-100 and 0.1% sodium citrate solution. The reaction solution from the kit was then added, before addition of SlowFade Gold antifade mountant with DAPI (Life Technologies) and observation with a BX51 fluorescence microscope (Olympus). Bright field and fluorescence images were acquired using the fluorescence imaging system Cell^A^ (Olympus).

### Reverse transcription quantitative PCR (RT-qPCR)

For RNA extraction, the RNeasy Plus mini kit was used (Qiagen, Courtaboeuf, France). Cells were harvested by centrifugation at 600xg for 10min and lysed with the RLT-Plus solution. After passing through a gDNA eliminator column, cells were washed with ethanol 70%, RW1 and RPE buffers. The concentration of the eluated RNAs was evaluated using a NanoVue Plus spectrophotometer (GE Healthcare, Vélizy-Villacoublay, France) before being aliquoted and conserved at -80°C. One-step reverse transcription was performed using the high capacity cDNA reverse transcription kit (Applied Biosystems, Foster City, CA, USA). RNA (10μL) was added to an equal volume of RT-PCR mix containing RT buffer, dNTPs, random primers and the multiscribe reverse transcriptase. Reverse transcription was performed using the following cycling conditions: 10min at 25°C, 120min at 37°C and 5min at 85°C. For quantitative PCR, 5μL of cDNA were added to 20μL of PCR mix containing Sybr Green I (Roche, France) and placed in a Light Cycler 480 with the following cycling conditions: Taq polymerase activation at 95°C for 10min and 45 cycles of amplification of 15sec at 95°C and 60sec at 60°C. The *kmp11* (Kinetoplastid Membrane Protein 11) gene was used as control, having the same level of expression in all the conditions used. Ratios of gene of interest/*kmp11* expression were calculated using the Pfaffl method where: ratio = (eff_gene_)^ΔCgene^(control-treated)/(eff_kmp11_)^ΔCqkmp11^(control-treated) with “eff” the efficiency, “control” the WT condition and ‘treated’ the death or autophagic condition. The PCR efficiency of the different oligonucleotide pairs was determined using the serial dilution method on the basis of a linear regression slope.

### Statistical analyses

For statistics, unpaired Student t-tests or Mann Whitney tests were done. Results were considered statistically significant when *p*<0.05. For significant differences, * means p<0.05, ** p<0.01 and *** p<0.001.

## Results

### Apoptotic drugs induce ovexpression of polyglutamylase genes

**Fig 1:**
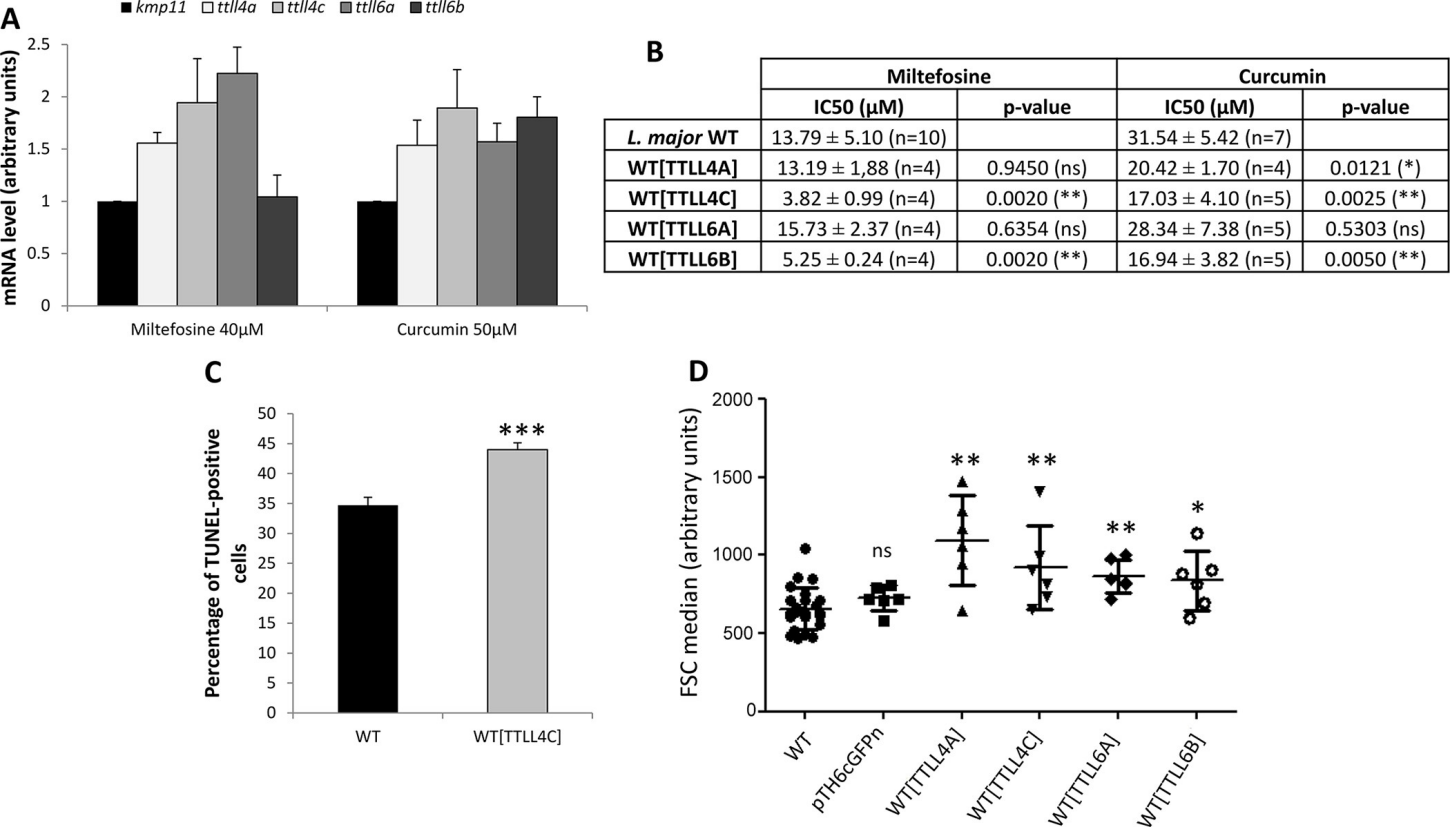
*Ttll* genes are overexpressed after cell treatment with miltefosine and/or curcumine and overexpression of polyglutamylases increased *Leishmania* miltefosine- and/or curcumin-induced cell death. (A) RT-qPCR quantification of the mRNA expression of *kmp11* (Kinetoplastid Membrane Protein, used as a control), and the four active polyglutamylases: *ttll4a*, *ttll4c*, *ttll6a* and *ttll6b*, after the addition of 40 μM of the pro-apoptotic drug miltefosine or 50 μM of curcumin for 24 hours. The expression was normalized to the expression in control conditions (without drug). Means ± sd from three independent experiments. (B) Miltefosine and curcumin IC50 for the WT cells and the cells overexpressing the polyglutamylases. The number of independent experiments (n) is mentioned in the figure. (C) Percentage of WT and TTLL4C overexpressing cells that are TUNEL-positive after the addition of 40 μM of miltefosine for 24 hours (means ± sd from three independent experiments). (D) Mean FSC median measured by flow cytometry of the WT cell line, the cells expressing the empty plasmid (pTH6nGFPc) and the cells overexpressing the polyglutamylases TTLL4A, TTLL4C, TTLL6A and TTLL6B, all treated with 40 μM of miltefosine (n = 25 for WT and n = 6 for the five other cells). The significant increase in FSC median indicates cell shrinkage, a hallmark of apoptosis. Mann Whitney test: ns: not significant, *: p<0.05, **: p<0.01, ***: p<0.001.

Four polyglutamylases have been identified as active in *L*. *major*: TTLL4A, TTLL4C, TTLL6A and TTLL6B [18]. In order to gain insight into the relationship between cell death and the PTM polyglutamylations, we monitored their expression by RT-qPCR in normal and death conditions. To induce *Leishmania* cell death, we added anti-*Leishmania* drugs previously described as regulated cell death-inducing drugs: miltefosine and curcumin [19,25,32]. These drugs notably induce growth inhibition, decrease in metabolic activity, cell rounding, cell shrinkage, calcein-positivity and TUNEL-positivity [19]. As shown in Fig 1A, the apoptotic drug miltefosine induced overexpression of the *ttll4a*, *ttll4c* and *ttll6a* genes, expression of these genes being 1.5 to 2.2 times higher than the expression of the housekeeping gene *kmp11* in death conditions in comparison to normal conditions. We note that the *ttll6b* gene is expressed at very high levels in *L*. *major*, as previously evaluated by Northern blot [18] and RNAseq [33], which could explain the difficulty to identify increased levels of expression during miltefosine-induced *Leishmania* cell death. Curcumin induced overexpression of the four genes coding for active polyglutamylases (expression 1.5 to 1.9 times higher for the *ttll* genes than for the *kmp11* gene) (Fig 1A). This indicates that polyglutamylase genes were overexpressed during *Leishmania* miltefosine- and curcumin-induced cell death.

### Overexpression of polyglutamylases induces *Leishmania* regulated cell death

We transfected *L*. *major* cells independently with vectors containing one each of the four active polyglutamylases, allowing the episomal expression of recombinant GFP-proteins and so overexpression of the corresponding TTLL. This overexpression induced no change concerning cell proliferation or cell survival in the absence of drugs, as shown on the growth curves in the S1 Fig. We carried out an MTT assay in order to determine the miltefosine and curcumin IC50 for each cell line, that is to say the drug concentration for which 50% of the cells are dead in comparison with control cells. As seen in Fig 1B, the miltefosine IC50 was significantly lower in cells overexpressing the polyglutamylases TTLL4C or TTLL6B, in comparison with the WT cells. Additionally, the curcumin IC50 was significantly lower in TTLL4A-, TTLL4C- and TTLL6B-overexpressing cells. Therefore, the overexpression of these polyglutamylases induced a higher sensitivity to miltefosine and curcumin.

In order to define the type of cell death process induced in TTLL overexpressing cells, we measured the percentage of apoptotic cells in each cell line, after miltefosine cell death induction. For this, we carried out a TUNEL assay. This technique, that evaluates DNA fragmentation, clearly identifies *Leishmania* apoptosis while calcein cannot be used in GFP-fluorescent cells [19]. We observed that TTLL4C overexpression induced a significant increase in the percentage of TUNEL-positive cells after the addition of miltefosine for 24 h (Fig 1C). However, no significant differences in the percentage of dead cells could be detected when the other three active polyglutamylases were overexpressed (S2 Fig). We also measured the Forward Scatter (FSC) by flow cytometry, an increase in FSC indicating cell shrinkage, which is a hallmark of *Leishmania* apoptosis [19]. As shown in Fig 1D, a significant increase in FSC was observed after miltefosine addition when any of the four different polyglutamylases was overexpressed, while the empty plasmid (pTH6cGFPn) induced no change in FSC. The fact that overexpression of all TTLL induced FSC increase after treatment with miltefosine while only TTLL4C appeared involved in *L*. *major* apoptosis according to the TUNEL assay could be explained by the fact that flow cytometry (for evaluating FSC) is more sensitive than fluorescence microscopy used for the TUNEL assay.

### CCP5A and CCP5B are deglutamylases that induce a flagellum length decrease and cell cycle defects

**Fig 2:**
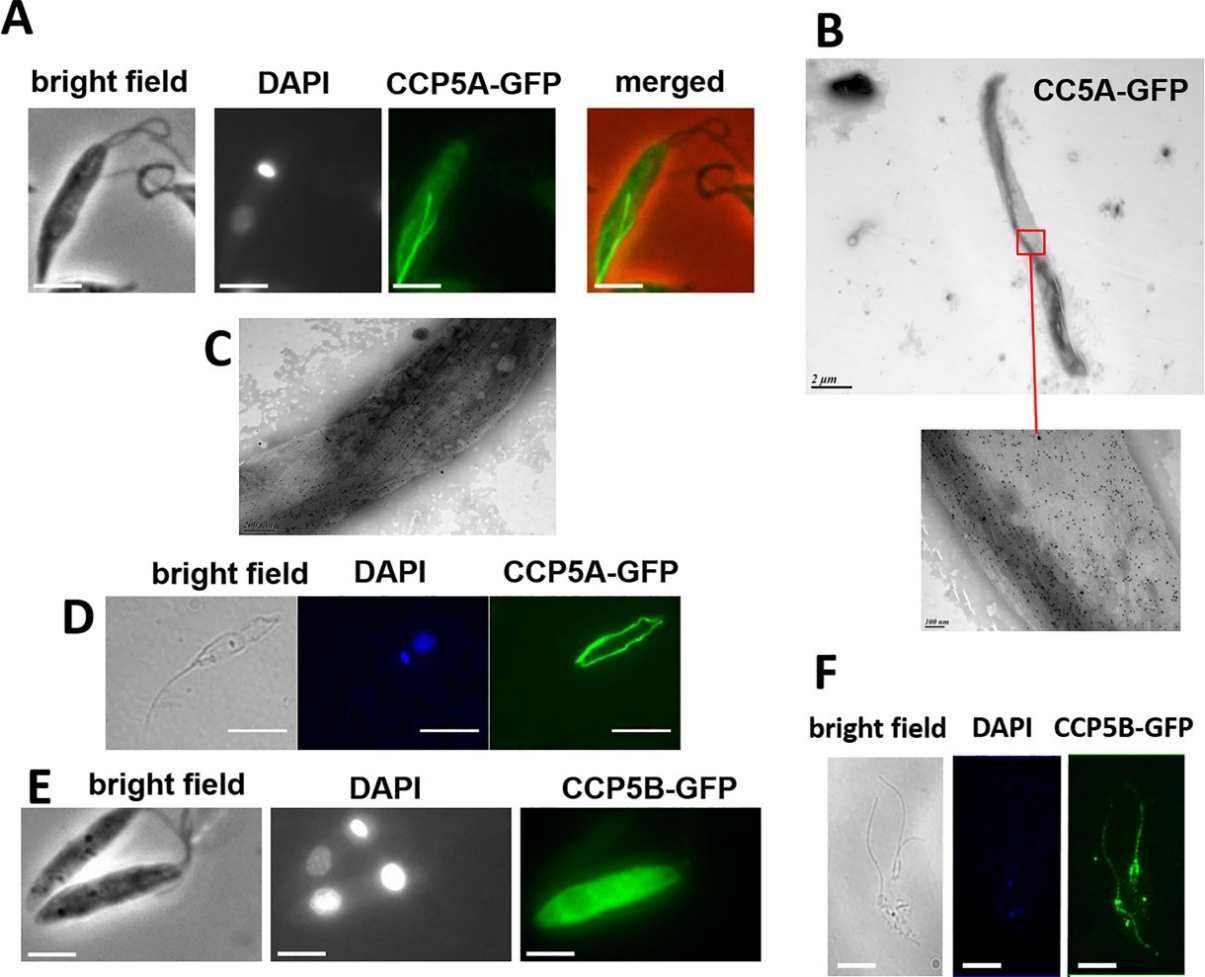
Cellular localization of CCP5A and CCP5B. (A) Fluorescence microscopy showing localization of CCP5A after fusion of the protein with GFP. CCP5A-GFP labeled filament-like structures in the cell body (bar = 5μm). (B) Immuno-electron microscopy of a cell expressing CCP5A-GFP. Filament-like structures were labeled with sometimes increased labeling within the cell. The lower panel is a magnification of the above square. (C) Another immuno-electron microscopy of a cell expressing CCP5A-GFP, showing strong label of a filament-like structure. (D) Localization of CCP5A-GFP after cytoskeleton extraction. The GFP-positive filament-like structure was still observed (bar = 5 μm). (E) Localization of CCP5B after fusion of the protein with GFP, showing labelling of the whole cell body (bar = 5μm). (F) CCP5B-GFP fluorescence after cytoskeleton preparation. CCP5B was localized at the flagellum and at the base of the flagellum (bar = 5μm).

Tubulin deglutamylases are members of the M14 zinc carboxypeptidase protein family. By using *in silico* GeneDB database (www.genedb.org), we identified two proteins: LmjF.34.2810 and LmjF.36.4030, that we named, respectively, CCP5A and CCP5B for their homology with the mammal CCP5 [18]. The study of their localization after episomal fusion with the green fluorescent protein (GFP) indicated that CCPP5A-GFP labelled filament-like structures in the cell body as visualized by fluorescence (Figs 2A and 4A and S3). These filament-like structures were often seen in rounded cells, as shown in S3 Fig. Immuno-electron microscopy indicated that the overexpression of CCP5A by transfection with CCP5A-GFP induced the appearance of a darker filament-like structure when negatively stained with Nanovan compared to the rest of the cell and that sometimes showed increased immunolabelling within the cell (Fig 2B and 2C). The filament-like structures were always present after cytoskeleton extraction, as seen by fluorescence microscopy (Fig 2D). Interestingly, when the CCP5A protein was tagged *in situ* by fusion of the endogene with the mNeonGreen sequence by CRISPR/Cas9, no filament-like structure was observed. On the contrary, CCP5B localized in the whole cell (Fig 2E). CCP5B-GFP was also found on the flagellum and at the base of the flagellum as shown after cytoskeleton extraction (Fig 2F).

**Fig 3:**
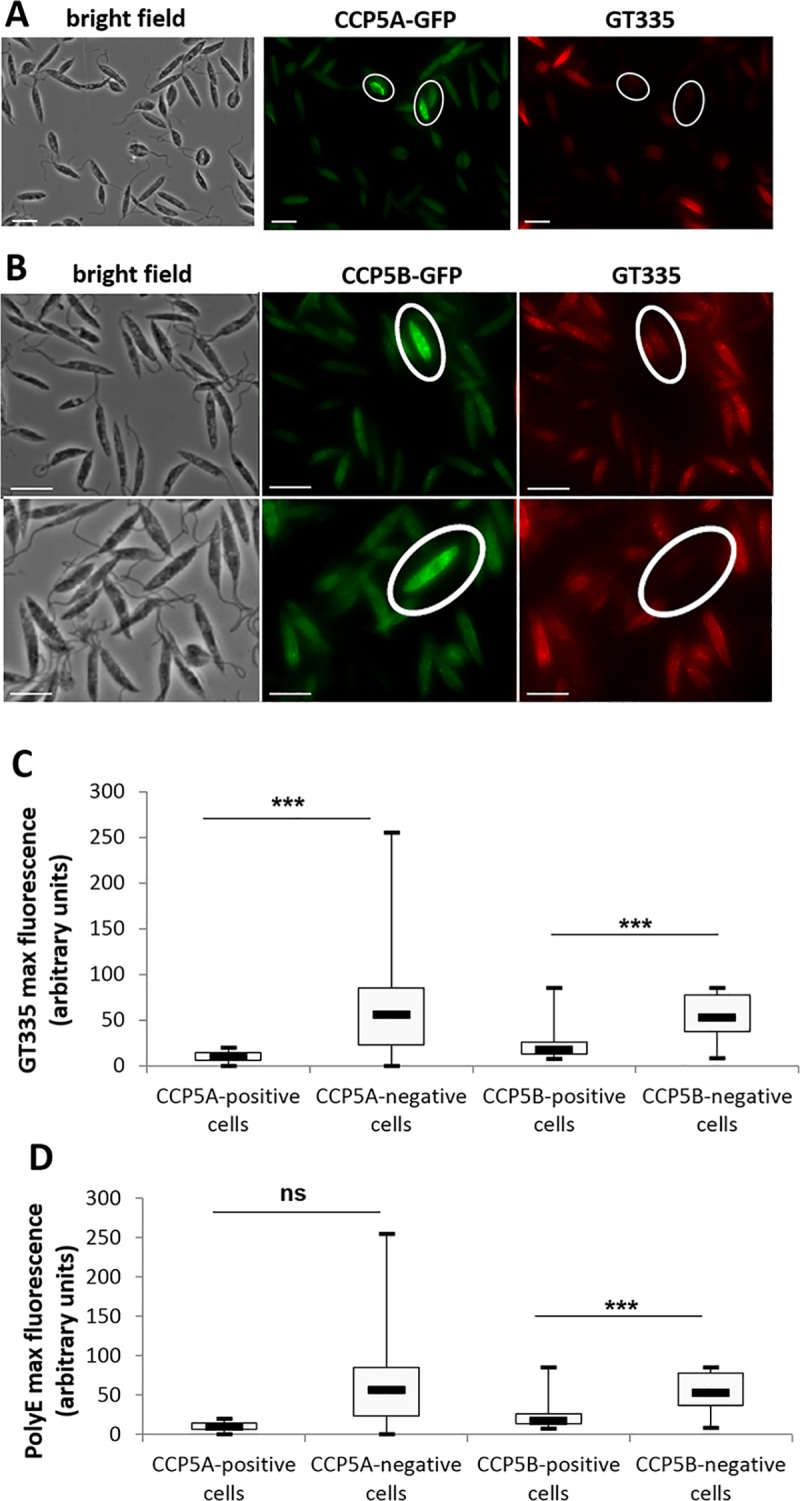
CCP5A and CCP5B are deglutamylases. (A) Immunofluorescence assay: CCP5A-GFP fluorescence in green and GT335 immunofluorescence in red (Texas Red). GT335 is a monoclonal antibody that recognizes all forms of polyglutamylated tubulin independently of the length of the polyglutamate side chain [34]. Here we show that cells overexpressing CCP5A (circled cells) were poorly stained with GT335, indicating CCP5A deglutamylase activity (bar = 10μm). (B) Immunofluorescence assay: CCP5B-GFP fluorescence (green) and GT335 immunofluorescence (red). The deglutamylase activity of CCP5B was shown by the poor GT335 labelling of cells clearly overexpressing CCP5B (circled cells) (bar = 10μm). (C) Quantification of the maximum of GT335 fluorescence, in arbitrary units, in CCP-positively (n = 17) and negatively (n = 151 for CCP5A and n = 99 for CCP5B) labeled cells. (D) Quantification of the maximum of PolyE fluorescence, PolyE being a polyclonal antibody recognizing long glutamate side chains. t-test: ns = not significant, ***: p<0.001.

To confirm the enzymatic activity of the CCP proteins, we carried out an immunofluorescence assay with GT335, a monoclonal antibody that recognizes all forms of polyglutamylated tubulin independently of the length of the polyglutamate side chain [34]. As previously demonstrated, in *Leishmania*, microtubules are intensely glutamylated at all stages of the cell cycle [18]. However, cells expressing CCP5A-GFP or CCP5B-GFP (circled in white in Fig 3A and 3B) were not labelled with the glutamylation specific antibody GT335 (Fig 3A and 3B, respectively). This deglutamylation in cells highly expressing CCP was confirmed by quantifying the maximum of GT335 fluorescence in CCP-positively and negatively stained cells: the maximum of GT335 fluorescence was significantly lower in CCP5A or CCP5B highly labelled cells in comparison to non-labelled cells (Fig 3C). To evaluate whether CCP5A and CCP5B remove one glutamate at the branching point or long side chains of glutamates, we carried out an immunofluorescence assay with PolyE, a polyclonal antibody that recognizes side chains of at least three glutamates long [35]. Fig 3D shows that the maximum of PolyE fluorescence was significantly lower in CCP5B highly labelled cells in comparison to non-labelled cells, suggesting that CCP5B removes glutamates at branching points and also from long side chains. On the contrary, the absence of significant difference in the PolyE labelling between cells highly expressing or not CCP5A suggests that CCP5A does not remove long glutamate side chains.

**Fig 4:**
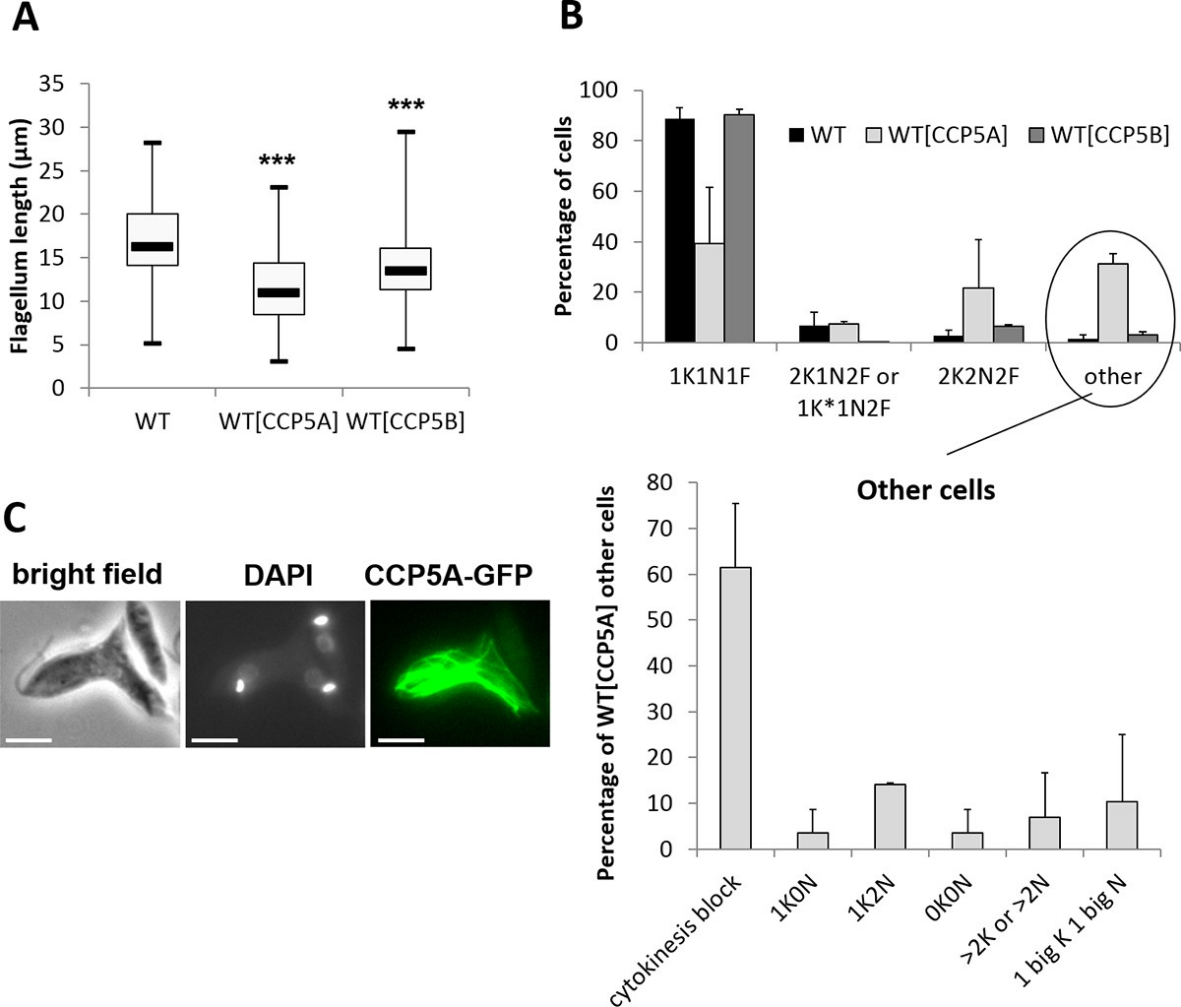
Overexpression of CCP5A and CCP5B induced flagellum length decrease and cell cycle defects. (A) Box plots representing flagellum length of WT, CCP5A overexpressing and CCP5B overexpressing cells. Minimum 40 cells were analyzed. The thick line inside each box represents the median value; the lower and upper edge of each box indicate the 25^th^ and 75^th^ percentiles, respectively; the lower and upper whiskers (ends of the box arms) represent the minimum and maximum, respectively. t-test: *: p<0.05, **: p<0.01, ***: p<0.001 (B) Cell cycle configuration of WT cells and cells overexpressing CCP5A or CCP5B. The lower panel corresponds to the configuration of the abnormal (other) cells. When CCP5A was overexpressed, we noted the appearance of abnormal cells, consisting essentially of cells blocked in cytokinesis (about 20% of the total population). The overexpression of CCP5B induced no obvious cell cycle defect (K = Kinetoplast; K* = Kinetoplast in replication or in G2 phase prior to segregation; N = Nucleus; F = Flagellum). (C) Fluorescence microscopy showing the localization of CCP5A as filament-like structures in a cell blocked in cytokinesis (bar = 5μm).

We noted that the overexpression of CCP5A and CCP5B, due to the episomal expression of the corresponding protein fused to the GFP, induced a significant decrease of flagellum length (Fig 4A). Furthermore, the overexpression of CCP5A induced severe cell cycle defects with the appearance of abnormal cells, as compared to the classical dividing *Leishmania* forms described by Ambit *et al*. [36], including about 20% of multinucleated cells apparently unable to terminate cytokinesis as exemplified by the description “cytokinesis block” in Fig 4B. Such abnormal cells are shown in Fig 4C and in S3 Fig, the filament-like structures being often found in cells blocked in cytokinesis. On the contrary, overexpression of CCP5B did not induce mitotic abnormalities (Fig 4B).

### Overexpression of deglutamylases inhibits regulated cell death

**Fig 5:**
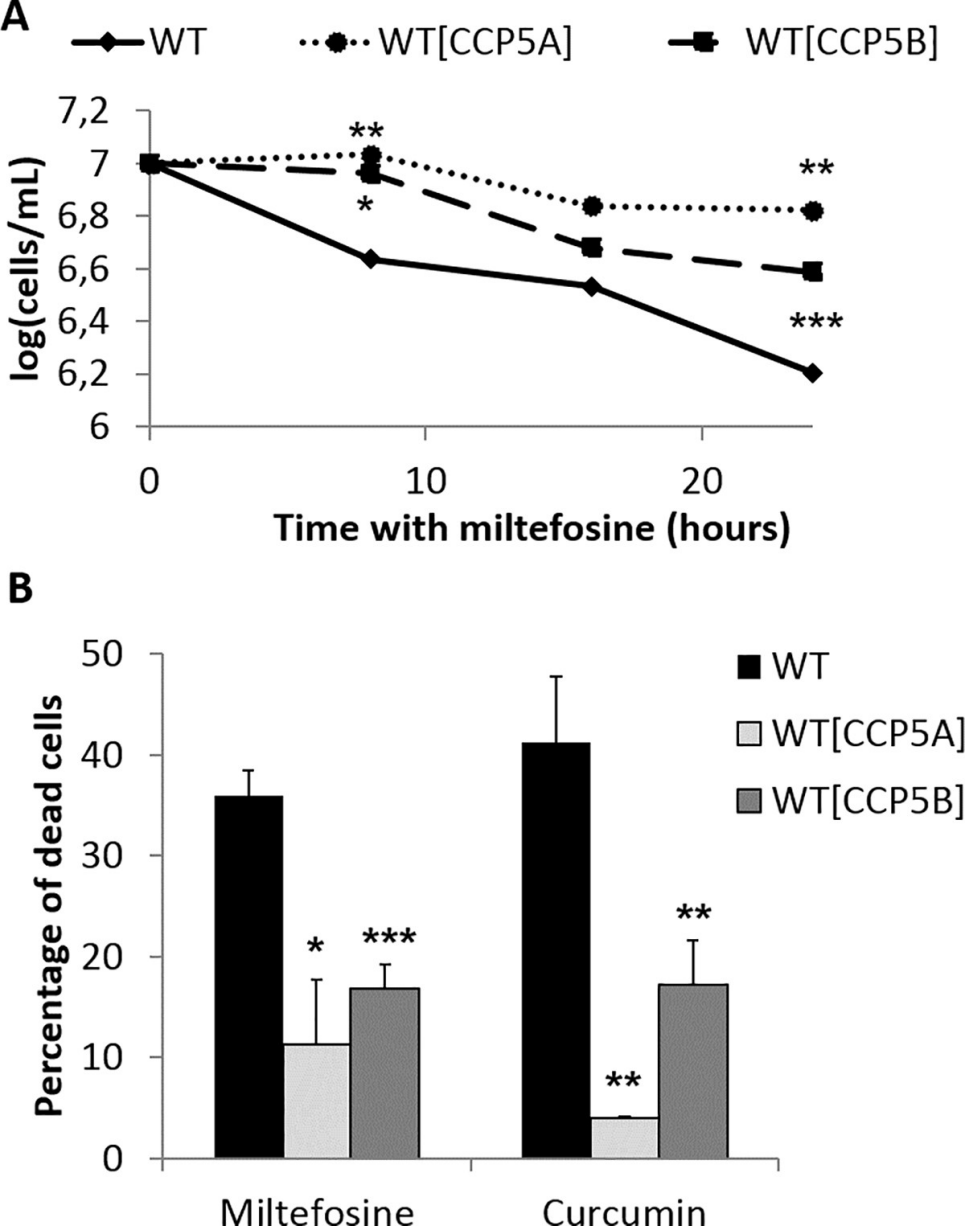
Overexpression of CCP5A and CCP5B inhibited RCD. (A) Growth curve of WT, CCP5A and CCP5B overexpressing cells after the addition of 40μM of miltefosine (means from a minimum of three independent experiments). The growth of the overexpressing cells was compared to the growth of the WT cells, in death conditions. (B) Percentage of dead (TUNEL-positive and anucleated) WT, CCP5A and CCP5B overexpressing cells after the addition of 40μM of miltefosine or 50 μm of curcumin: means ± sd from three independent experiments. The percentage of dead cells significantly decreased in each deglutamylase overexpressing cells in death conditions. Means ± sd from a minimum of three independent experiments. Student t-tests: *: p<0.05, **: p<0.01, ***: p<0.001.

Overexpression of CCP5A and CCP5B, owing to the episomal expression of the recombinant GFP-CCP protein, induced significant changes in the growth curve when cells were cultivated with 40μM of miltefosine, while the growth was similar to WT cells in the absence of drug. Indeed, CCP5A and CCP5B overexpressing cells had a significantly reduced death rate when cultivated with miltefosine (Fig 5A). This growth difference was linked to a decrease in the percentage of TUNEL-positive cells, compared to WT cells (Fig 5B). The reduction in the percentage of apoptotic cells when CCP were overexpressed was also observed in the presence of curcumin (Fig 5B). As a consequence, overexpression of the deglutamylases inhibited miltefosine and curcumin-induced RCD.

### Deglutamylase genes are overexpressed during autophagy

**Fig 6:**
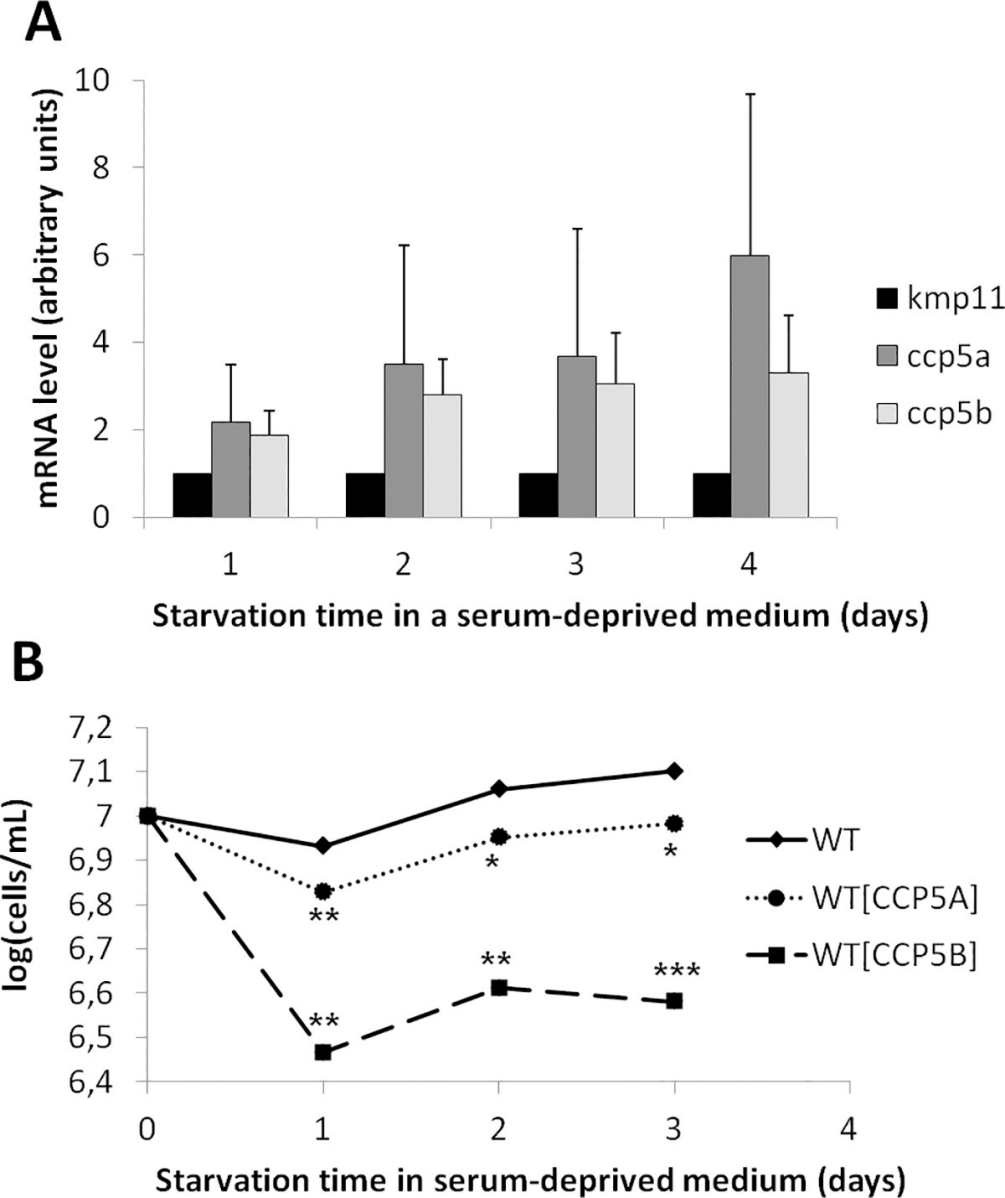
Deglutamylase genes are overexpressed during *L*. *major* autophagy. (A) RT-qPCR quantification of *kmp11* (Kinetoplastid Membrane Protein, used as a control), *ccp5a* and *ccp5b* mRNA expression, after culture of WT cells in a serum-deprived medium (means ± sd from a minimum of three independent experiments). We noted overexpression of *ccp5a* and *ccp5b* when cells were cultivated in a serum-deprived medium. (B) Growth curve of WT, CCP5A and CCP5B overexpressing cells cultivated in a serum-deprived medium (means from a minimum of three independent experiments). Significant growth defects could be observed in starvation conditions when CCP5A or CCP5B when overexpressed. Student t-test: *: p<0.05, **: p<0.01, ***: p<0.001.

Since autophagy is a process allowing the cell surviving nutrient depletion, that is closely linked to RCD [37], we have studied the relationships between autophagy and (de)glutamylation. By carrying out RT-qPCR experiments, we observed that the *ccp5a* and *ccp5b* genes were overexpressed when the cells were cultivated in a serum-deprived medium, therefore in autophagic conditions: the expression of these genes was 2 to 6 times higher than expression of the control gene *kmp11*, in autophagic conditions in comparison to normal conditions (Fig 6A). In addition, overexpression of CCP5A or CCP5B by transfection of *L*. *major* cells with GFP-tagged proteins induced significant growth defects when cells were cultivated in a serum-deprived medium (Fig 6B). These defects were not linked to apoptosis since no increase in the percentage of TUNEL-positive cells was observed in cells overexpressing CCP5A or CCP5B during *Leishmania* autophagy (S4 Fig).

## Discussion

*Leishmania* are unique unicellular eukaryotes. Indeed, beside their high phylogenetic distance from other eukaryotes traditionally studied [35], they present several molecular and cellular originalities. For instance, microtubules form a corset covalently linked to the plasma membrane and covering the whole cell. Furthermore, the actin network appears structurally and functionally different from other eukaryotic actins [1]. Or, in terms of cell death, while an apoptotic phenotype has been characterized in *Leishmania*, the pathways remain largely unknown, being devoid of key mammal cell death proteins such as caspases, cell death receptors, or anti- or pro-apoptotic molecules [29]. As a consequence, *Leishmania* appears as a model of choice to study eukaryotes, highlighting original processes.

During *Leishmania* cell death, important cytoskeleton modifications appear (cell rounding up, decrease of flagellum length…) [36]. In order to explain these cytoskeleton modifications, we have studied PTM during *Leishmania* cell death. We have shown a link between polyglutamylase expression and cell death in *Leishmania*. Indeed, during *Leishmania* cell death induced by the addition of the pro-apoptotic drugs miltefosine and curcumin, polyglutamylase genes were overexpressed. Furthermore, overexpression of some polyglutamylases renders the cells more sensitive to cell death induced by miltefosine or curcumin. The overexpression of the polyglutamylases also induced cell shrinkage, a hallmark of apoptosis. Last, the importance of polyglutamylases in RCD was demonstrated by an excess of apoptosis when TTLL4C was overexpressed. We could not rule out the involvement of TTLL other than TTLL4C in RCD entry, for instance TTLL6A whose gene is highly overexpressed during miltefosine-induced *L*. *major* cell death. However, the presence and function of other TTLL could not be detected owing to their possible low episomal expression levels with the pTH6GFP vector used, relative to the endogenous proteins. For instance, *ttll4a* and *ttll6b* are highly expressed in *Leishmania* cells, as previously shown [18,33], which could render the visualization of the consequences of the overexpression of the proteins difficult. We could also see no consequence of TTLL overexpression owing to the necessity of concomitant overexpression of different TTLL, or to the lack of an activation step or cofactors, as already suggested [10,13]. A good example of the complexity of activation is observed with TTLL1, which in higher eukaryotes is known to be active only as part of a multiprotein complex [10].

The nature of the TTLL substrates remains to be discovered. Even if a clear polyglutamylase activity has been described for TTLL4A and TTLL6B against tubulin and also non-tubulin substrates, no activity has been recorded for TTLL4C and TTLL6A against tubulin and only a slight activity has been recorded against the non-tubulin substrate NAP1 [18]. However, the experimental assay used in this previous article did not include cell death conditions. Yet, overexpression and RNA interference-based knockdown of the four active polyglutamylases have no or very little effect on cell growth in normal conditions [18]. We can thus hypothesise that the polyglutamylases must be activated by pro-apoptotic drugs in order to induce excessive polyglutamylation in the cell, and so to induce RCD. We note that in this work, the overexpression of the different genes was obtained by the episomal expression of the gene fused to the sequence of the GFP. Therefore, we cannot rule out an effect from the GFP tag in the consequences of gene overexpression.

We have also identified, for the first time in *Leishmania*, deglutamylases, that we named CCP5A and CCP5B for their homology with the mammal CCP5. In an original manner for CCP5 proteins [12], CCP5B seems to remove not only glutamates at branching points but also long glutamate side chains. CCP5B localized in the whole cell, a CCP5B-GFP labeling remaining at the flagellum and at the base of the flagellum after cytoskeleton extraction. Concerning CCP5A, its localization appeared more peculiar. Indeed, when the protein was overexpressed by the episomal expression of the GFP recombinant protein, we observed GFP-positive filament-like structures still present after cytoskeleton extraction, mainly in rounded cells that seemed blocked in cytokinesis. On the contrary, when the endogene was fused *in situ* with the mNeon Green sequence, the filament-like structures were not observed, the mNeon Green labeling being distributed in the whole cell. This peculiar localization is reminiscent of the localization of actin in *Leishmania*. Actin, while highly abundant in *Leishmania*, presents unconventional properties compared to mammal actin, among which polymerization conditions, different ATPase and DNase I activity or binding to phalloidin or Latrunculin B [37]. Its *in situ* localization revealed that it is mainly present as granules and possibly as patches and short filaments [1]. On the contrary, when overexpressed, *Leishmania* actin organizes as long cables/bundles [38]. The similarity of localization between actin and CCP5A suggests that CCP5A deglutamylates actin, inducing the formation of high amounts of filamentous actin that organizes as bundles. To strengthen this hypothesis, we identified in the CCP5A sequence, from amino acids 488 to 494, a putative actin-binding site (SRKRHPA) similar to the one of coronin (SRFRHST), which is a protein associated with the filament-like structures of actin in *Leishmania* promastigotes [39]. Actin in Trypanosomatids has been described as required in vesicular transport during endocytosis [40].

The episomal expression of CCP-GFP proteins induced the inhibition of *Leishmania* apoptosis induced by miltefosine and curcumin, confirming the link between deglutamylases/polyglutamylases and *Leishmania* cell death. Since RCD is paradoxically closely linked to the cell survival process autophagy [37], we studied the relationships between autophagy induced by serum deprivation and deglutamylation. We observed that the deglutamylase genes *ccp5a* and *ccp5b* were highly transcribed during serum deprivation. Furthermore, the episomal expression of CCP-GFP induced growth defects during autophagy induced by serum deprivation. We thus hypothesized that an autophagic stimulus would induce overexpression of CCP5A and CCP5B and that, owing to their cytoskeleton localization and to the consequences of their overexpression, CCP5A and CCP5B would deglutamylate actin but also microtubules, notably microtubules of the flagellum, and induce changes in the interaction of microtubules with microtubule-modifying proteins. This could induce loss of mobility observed during autophagy [19]. This hypothesis is consistent with the idea that tubulin deglutamylases play important roles in cilia function in higher eukaryotes [41]. A good example of this is *Caenorhabditis elegans*, where the tubulin deglutamylases CCPP-1 and CCPP-6 localize to cilia and mutation in *ccpp-1* causes excessive accumulation of KLP-6 kinesin and polycystin-2 in cilia and an increase in the transport rate of OSM-3/KIF17 on axonemal microtubules [11,42]. In zebrafish, expression of the deglutamylase genes *ccp2*, *ccp5* and *ccp6* is strongly enriched in ciliated cell types [43]. Furthermore, *ccp5* deficiency induces cilia microtubule hyper-glutamylation and motility defects without affecting overall cilia length [43]. A cross-talk between autophagy and cilia has also been demonstrated: signaling from the cilia can recruit the autophagic machinery to trigger autophagosome formation and autophagy induces ciliogenesis by controlling the level of ciliary proteins [44,45]. In *L*. *major*, this cross-talk could be linked to the deglutamylases CCP5A and CCP5B.

**Fig 7:**
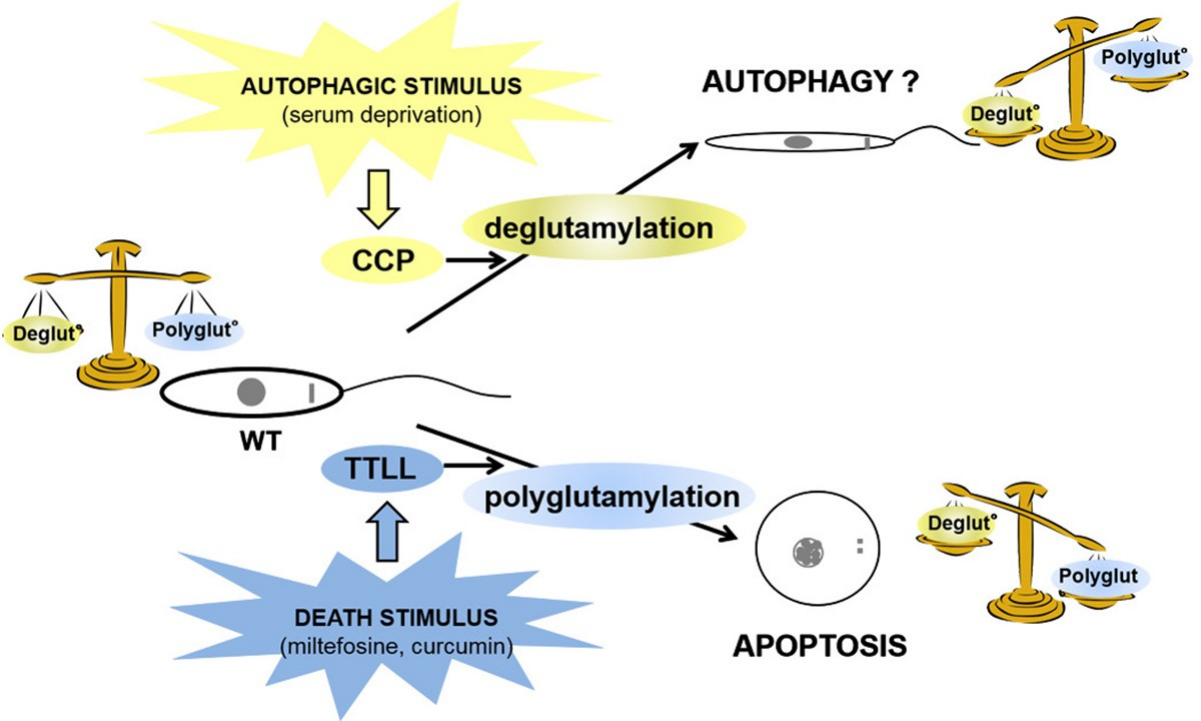
Model: relationship between cellular deglutamylation/polyglutamylation balance, RCD and autophagy. In the model based on the results obtained here, we suggest that a balance is established in WT cells between deglutamylation and polyglutamylation. An autophagic stimulus like serum deprivation induces overexpression of deglutamylases, inducing imbalance towards severe deglutamylation, responsible for the autophagic survival phenotype. On the contrary, a death stimulus such as miltefosine or curcumin induces severe polyglutamylation through activation of TTLL, inducing RCD.

We illustrated this hypothesis in the model in Fig 7 where a death stimulus in *Leishmania* would induce polyglutamylation, at the origin of apoptosis. On the contrary, an autophagic stimulus would induce overexpression of deglutamylases and therefore microtubule and/or other protein deglutamylation, inducing modifications mainly of the flagellum, at the origin of the autophagic phenotype and thus cell survival.

As a conclusion, through using *Leishmania*, we highlighted a link between polyglutamylases and cell death, suggesting the importance of the polyglutamylation/deglutamylation balance in the cell cycle. Imbalance would induce either apoptosis if polyglutamylation took precedence or autophagy if deglutamylation was prioritized. Even if the kinesins, microtubule-associated proteins or microtubule-severing factors interacting with microtubule modifications have to be identified in order to complete the proposed model, this work emphasized the role of PTM as essential regulators of protein function. This role has already been described, notably concerning microtubules, tubulin PTM having been linked to several pathologies: cilia-related disorders, neurodevelopmental and neurodegenerative disorders, bleeding disorders, cardiac diseases and cancer [41]. However, the importance of cytoskeleton modifications had not been emphasized in infectious diseases.

## Supporting information

S1 Fig Growth curves of WT cells and cells expressing the different TTLL-GFP. The cells expressing the recombinant TTLL had no growth defect in comparison to WT cells.
(TIF)

S2 Fig Percentage of TUNEL-positive WT and polyglutamylase-overexpressing cells after the addition of 40μM of miltefosine for 24 hours. Means ± sd from three independent experiments. No significant difference was observed between the overexpressing and the WT cells.
(TIF)

S3 Fig Fluorescence microscopy showing, in different *L. major* cells, localization of CCP5A after fusion of the protein with GFP and localization of tubulin with an anti-α-tubulin antibody (bar = 5 μm). (TIF)

S4 Fig Percentage of TUNEL-positive WT, CCP5A or CCP5B overexpressing cells after culture in autophagic conditions (PBS). Means ± sd from minimum three independent experiments. Student t-test: ns: not significant, **: p<0.01.
(TIF)
