# Supplementary figures and images for: (De)glutamylation and cell death in Leishmania parasites
Source: PLoS Negl Trop Dis. 2019 Apr 24;13(4):e0007264. doi: 10.1371/journal.pntd.0007264 (PMC6502457; doi:10.1371/journal.pntd.0007264)

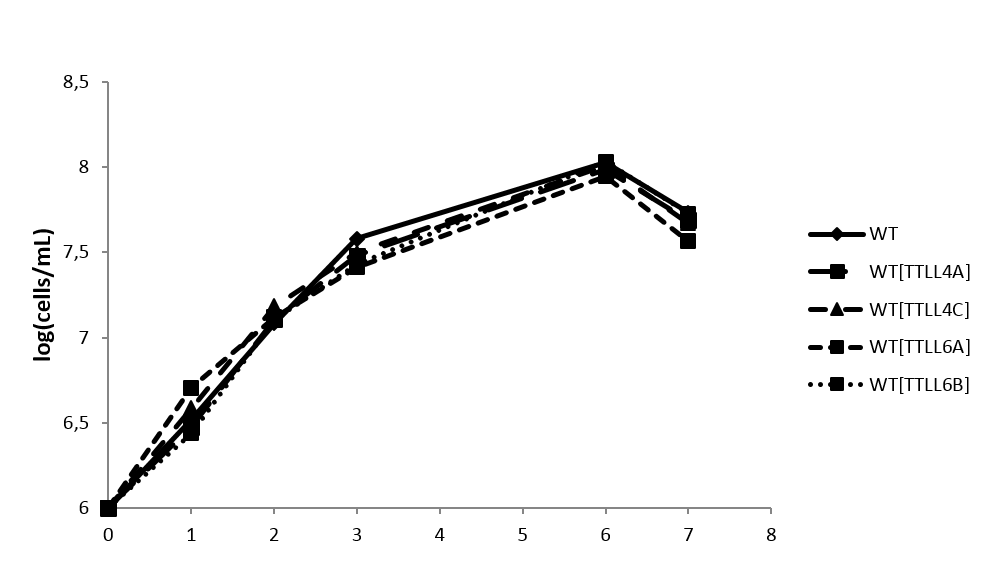

Supplement: S1 Fig — The cells expressing the recombinant TTLL had no growth defect in comparison to WT cells. (TIF) [file pntd.0007264.s001.tif]

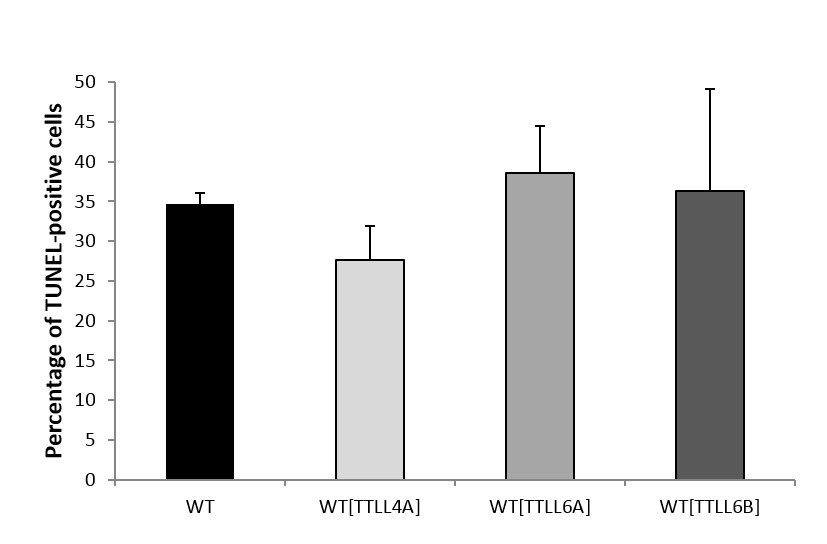

Supplement: S2 Fig — Means ± sd from three independent experiments. No significant difference was observed between the overexpressing and the WT cells. (TIF) [file pntd.0007264.s002.tif]

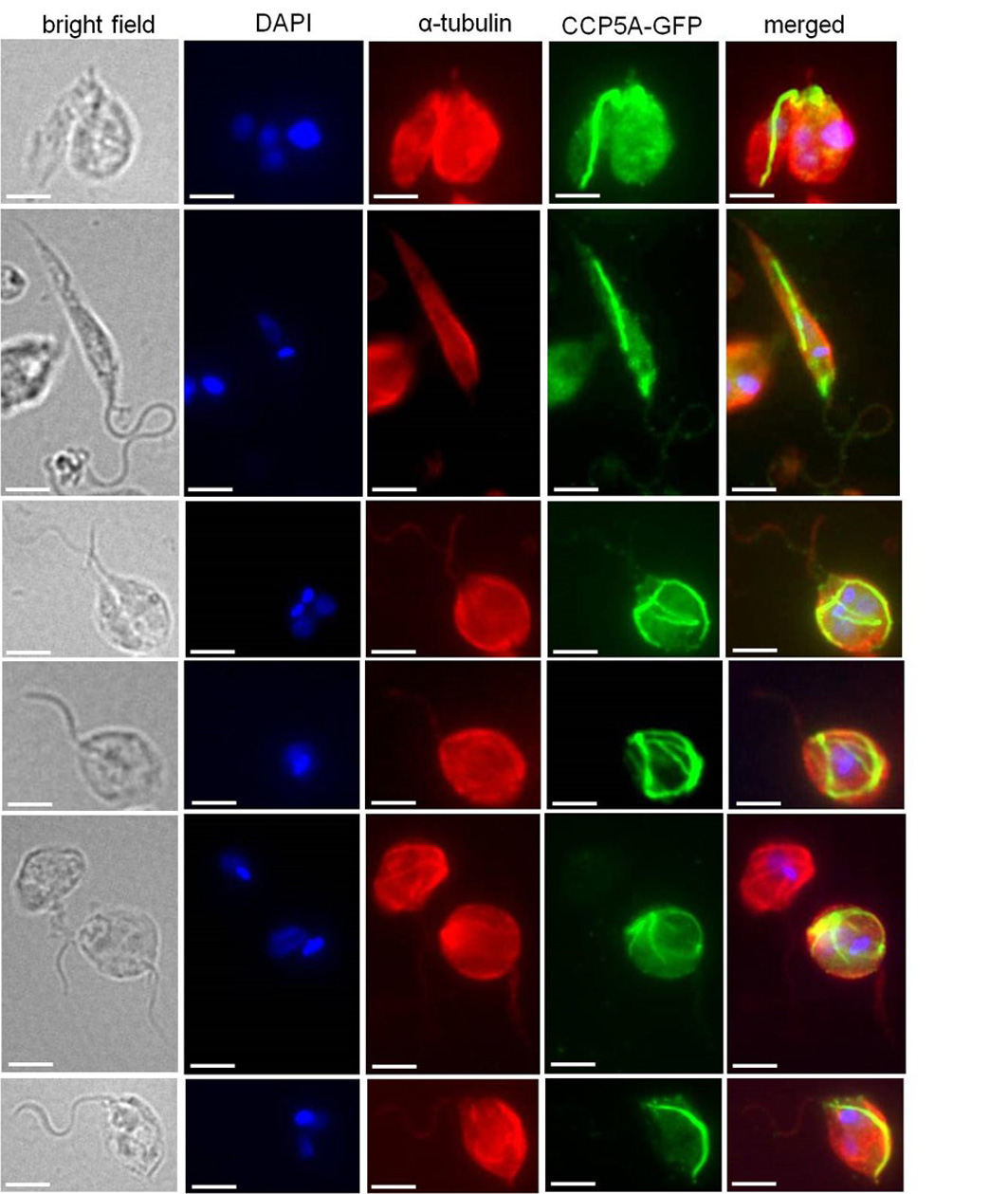

Supplement: S3 Fig — (TIF) [file pntd.0007264.s003.tif]

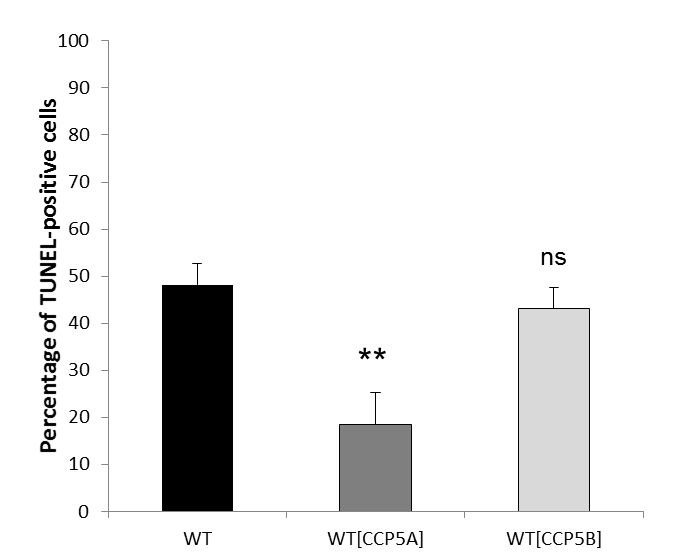

Supplement: S4 Fig — Means ± sd from minimum three independent experiments. Student t-test: ns: not significant, **: p<0.01. (TIF) [file pntd.0007264.s004.tif]
